# Nutritional composition of various insects and potential uses as alternative protein sources in animal diets

**DOI:** 10.5713/ab.21.0447

**Published:** 2022-02-01

**Authors:** Assar Ali Shah, Pajaree Totakul, Maharach Matra, Anusorn Cherdthong, Yupa Hanboonsong, Metha Wanapat

**Affiliations:** 1 Tropical Feed Resources Research and Development Center (TROFREC), Department of Animal Science, Faculty of Agriculture, Khon Kaen University, Khon Kaen 40002, Thailand; 2 Department of Entomology and Plant Pathology, Faculty of Agriculture, Khon Kaen University, Khon Kaen 40002, Thailand

**Keywords:** Alternative Protein Source, Animal Feed, Bioactive Nutrients, Insect Farming, Insect Meal

## Abstract

The aim of the present investigation is to determine the nutritional composition of various insects and their potential uses as alternative protein sources in animal diets. The feeding industry requires production systems that use accessible resources, such as feed resources, and concentrates on the potential impacts on production yield and nutritional quality. Invertebrate insects, such as black soldier flies, grasshoppers, mealworms, housefly larvae, and crickets, have been used as human food and as feed for nonruminants and aqua culture while for ruminants their use has been limited. Insects can be mass-produced, participating in a circular economy that minimizes or eliminates food- and feed-waste through bioconversion. Although the model for formula-scale production of insects as feed for domestic animals has been explored for a number of years, significant production and transformation to being a conventional protein resource remains to be deeply investigated. This review will focus on the nutritional composition of various insects and their potential use as alternative protein sources, as well as their potential use to promote and support sustainable animal production. Furthermore, nutritional compositions, such as high protein, lauric acid omega 6, and omega 3, and bioactive compounds, such as chitin, are of great potential use for animal feeding.

## INTRODUCTION

Insects have been addressed as one of the most promising alternative protein sources to solve the international dilemma of protein meal production for human food and animal feeds. The most significant benefit of insects over other protein sources is the low ecological charge of production that is necessary to satisfy the world protein requirements [1]. Recently, insects have been recognized as a significant potential resource of sustainable new raw resources for animal diets in several countries, perhaps the world. Primary insects encompass the nutritional requirements of animal feed in terms of nutrients and amino acid (AA) profiles and are part of the natural diets for some animal species [2]. From an ecological point of view, large-scale production of insects promises lower greenhouse gas emissions, the production of 1 kg of protein in smaller landfills, lower feed–food competition, a reduction in land use, and the conversion of organic supplements into high-value protein products [3]. The food and agriculture organization (FAO) [4] has extensively complied “edible insects” and stressed the highlights and challenges underpinning the research gaps and the ability to scale-up production and engage all stakeholders. Many insects have been used as animal diets, and the most promising insects are the black soldier fly (BSF), grasshopper mealworm, and housefly larva [5–8]. Schiavone et al [9] reported that the use of BSF (*Hermetia illucens*) larva at 5% dry matter (DM) intake successfully improved the growth rate, feed efficiency, carcass and immunological status (through chitin, lauric acid, and peptides) of broiler chickens. Specifically, the use of insects in bioconversion is a significant example of a new approach and a sustainable circular financial system [9,10].

Their high potential alternative as a feed component is associated with their short life cycle and, therefore, their ability to be reared on a large scale and at competitive trade prices compared to the other species proposed as animal feeds. The possibility of incorporating insect larvae, preppa, and fat in the diets of fish, poultry, and dairy animals has been investigated previously as part of the usual protein and fat sources (i.e., fish, oil, and soybean meal [SBM]) [2]. Beneficial results have been seen in mammal performance, gut strength characteristics, and product value. The use of insects as a diet supplement to achieve better intestinal performance has also gained significance. It contains bio-bacterial ingredients, such as antimicrobial peptides, lauric acids, and chitin immune-boosting properties [11].

The FAO estimated that there has been an increased demand for animal protein, with poultry meat accounting for the most significant increase in meat consumption. Thus, increased poultry meat production is expected and exacerbates the previous challenges of providing potentially costly reliable feed. Ingredients for animal feeds include fish oil, soybeans, seed cakes, and many additional grains [12]. Moreover, the international forum of insects for food and feed anticipates that the use of insects for food and feed in the European Union will increase from 500 tons in 2020 to more than 1 million tons in 2025, with 3 million tons by 2030 (larvae and adult). This positive market trend may reflect the profit that stakeholders receive by producing insects, which will increase consumer awareness of the adverse effects of animal feed production [13,14].

It is difficult to simplify the nutritional composition of edible insects, as more than 2,100 different species are eaten. Edible insects can offer nutrients and nutritional energy to meet the requirements of the animal and human body as part of a diverse diet [14]. Alternatively, the nutritional compositions of insects vary significantly between and within species, depending on the insect’s metamorphic stage, habitat, and diet. In addition, they are rich in proteins (i.e., AA, such as cysteine, lysine, methionine, and threonine), carbohydrates, fats, and several minerals (e.g., calcium, iron, zinc, and phosphorus), and essential vitamins (e.g., vitamin A, B complex, and vitamin C). Experiments have shown that insects are suitable for food, as they comprise 77% to 98% protein. Insect protein quality is also high in terms of essential amino acids (EAA) [1]. Van Huis [15] reported that insects, such as BSF, cricket, silk worm, housefly, yellow mealworm, grasshopper, and house fly maggots, have a potential use as protein meal in animal feeds. The EAA, such as arginine, lysine, and methionine, can enhance the utilization in chickens as well as in fish culture. Insects play an important and essential role as a protein-based feed to ensure food security. In addition to the nutritional value derived from high concentrations of protein and AA, insects contain high levels of fatty acids (FA), especially unsaturated FA.

Additionally, Ahmed et al [16] showed that edible insects (*Acheta domesticus*, *Brachytrupes portentosus*, *Gryllus bimaculatus*, and *Bombyx mori*) used at 25% of SBM in a concentrate supplement significantly improved (p<0.01) *in-vitro* fermentation (reduced CH_4_) and maintained degradability. In addition, other findings were similar to SBM treatments because of insects’ high CP, AA profiles, and FA (14% to 26%, esp unsaturated FA, omega 6 and 3).

Consequently, it appears appropriate to consider insect protein as a profitable feed source in the future. Conversely, there is little research on consumer and stakeholder perspectives on the use of insects in farm animals diets. Therefore, the article is arranged as follows. After the introduction, Section 1 presents an overview of the legislative structure to introduce a new source of protein in the animal husbandry industry. Section 2 details the chemical composition and nutritive value of various insects for use in animal feed. Section 3 includes an economical assessment of insect protein compared to other protein sources. Section 4 provides information on the current production status of insect farming. Section 5 outlines the advantages and limitations of using insects in animal diets. Section 6 includes the regulations for insect use as determined by the governments in Asia. Finally, the conclusion provides an outline of the present challenges and future investigations in this area.

## CHEMICAL COMPOSITION AND NUTRITIVE VALUE OF THE VARIOUS INSECTS FOR ANIMAL FEEDING

### Grasshoppers chemical composition and nutritive value

Grasshoppers can be grown within their natural habitats, such as farms, grasslands, paddocks, and wetlands [17]. Harvesting grasshoppers from these habitats can decrease the use of effective elements to control insects. In this way, these hurtful insects can be used in an economical and self-sustaining way, especially in developing countries, as a protein source in animal feed [5,8]. The grasshopper body has three main components: the head contains sensory structures, such as eyes, antennae, and mouth parts; the thorax has structures associated with mobility, such as the legs and wings; and the abdomen contains the digestive and reproductive structures [18].

Table 1 lists the average nutritive values of a grasshopper. On average, grasshoppers consist of DM (35.00%), crude protein (CP, 50.50%), crude fiber (CF, 15.30%), ash (6.40%) on DM basis. Furthermore, a grasshopper’s average mineral concentrations are as follows: calcium (Ca) 146.0 parts per million (ppm), phosphorous (P) 153.0 ppm, magnesium (Mg) 56.40 ppm, potassium (K) 344.0 ppm, zinc (Zn) 22.50 ppm, copper (Cu) 05.30 ppm, manganese (Mn) 01.40 ppm, and iron (Fe) 32.20 ppm. Many scientists have estimated the nutrients in grasshopper meal (GHM; Table 1). As noted by Makkar et al [5] and Khan [8], this nutritional information is extremely unpredictable. The CP concentration fluctuates between 29% to 77.1% depending on species, developmental phase, and processing technique. Ojewola et al [19] found that grasshoppers contained ash (9.97%), CF (2.38%), CP (28.13%), ether extract (EE, 4.18%), and gross energy (GE, 1,618 kcal/g), noted respectively on basis of DM. Alternatively, Makkar et al [5] and Khan [8] found the contents of grasshopper: ash (4.31%), CF (9.21%), CP (53.58%), EE (26.52%), and nitrogen-free extract (6.40%), noted respectively on basis of DM. The adult grasshopper mostly consists of chitin (8.73%), CP (65.42%), and fat (8.3%) based on DM [20]. Ghosh et al [21] found that in small horn grasshopper all EAA, such as cysteine (3.5%), isoleucine (2.85%), lysine (4.29%), leucine (2.53%), and threonine (3.75%), were found in large quantities, but arginine, tyrosine, histidine, methionine, tryptophan, and valine were found in small quantities. For AA, glutamic acid and glutamine quite were high in 30% of DM insects, followed by serine (6% DM) [20]. Five FAs, linoleic acid, lanolinic acid, oleic acid, palmitic acid, and stearic acid occurred in grasshoppers and contained about 89.6% total FA. The same researchers reported that certain minerals were present: Cu (4.36 mg/100 g DM), Fe (16.19 mg/100 g DM), Mg (84.84 mg/100 g DM), and Zn (17.34 mg/100 g of DM). Similarly, the following vitamins were found in high concentrations: vitamin B_3_ (29.59 mg/ 100 g DM), vitamin B_2_ (2.55 mg/100 g of DM), vitamin C (26.73 mg/100 g of DM), and vitamin A (0.12 mg/100 g of DM) [21–23]. Recently, Ssepuuya et al [23] found that grasshoppers (*Rasulia nitidula*) contained 36% to 40% CP, 2.5% to 3.2% carbohydrate, 41% to 43% EE, 2.6% to 3.9% ash, 11.0% to 14.5% CF and 900 to 2,300 μg/100 g total carotenoids based on DM.

### Grasshopper effect on ruminant animal digestibility

Insects are an increasingly attractive feed and food product, especially as a new and sustainable source of high-quality protein for animal production. So far, approximately 2,000 species of edible insects have been recognized. Although the nutritional value of insects varies from species to species either fresh or processed they are generally compared to beef or fish in many nutritional aspects. The protein content ranges from 350 to 700 g/kg of DM, and the quality of protein in edible insects is considered outstanding, as shown by the rich AA profiles and digestive properties. Notably, most properties that affect the nutritional composition of insects’ efficiency as feed depends on the synthesis of the food eaten by the insects [23,24]. Patton and Chandler [24] reported the *in-vivo* rumen digestibility by using grasshoppers, crab meal, cockroaches, and shrimp meal. The *in-vivo* digestibility system concerned a variation of the balanced nylon bag performance. The nylon bag was balanced inside the rumen of fistulated Jersey cows. The comparatively small solubility of the substances appears in the short time for 12 h incubation and the average rumen digestibility for the following insects was grasshoppers (66.5% of DM), shrimp meal (32.0% of DM), cockroaches (17.4% of DM), and crab meal (21.5% of DM). The *in-vitro* protein digestibility and vitamin substances of feather termites, green locusts, and brown locusts were determined with standard techniques. The investigation was performed on fresh, freshly dried, and toasted insect samples. There was no significant change to (p>0.05) protein digestibility in termite specimens, but a significant reduction in locust (p≤0.05) sample digestibility was seen during testing and drying. There was a significant decrease in riboflavin 4.18 mg/100 g in fresh termites, 2.76 mg/100 g in toasted lemons, 2.26 mg/100 g in fresh dried lemons, and 1.50 mg/100 g in toasted dry termites after processing [25].

Generally, protein decreased in relation to *in-vitro* digestibility, with the maximum protein digestibility found in fresh insect species. On the other hand, the digestibility waste in termites (*M. subhylanus*) for fresh (90.49% of DM), toasted (90.36% of DM), and dry/dried (90.13% of DM) samples was not significant. However, the decline was noticeable in the brown and green grasshopper samples [26]. Depending on the processing environment, high-temperature processing can decrease or enhance protein digestibility. Introduction to fluctuating heat can increase the digestibility of original proteins by opening the polypeptide chain and making the protein further sensitive to digestive enzymes [27,28]. In addition, when proteins are exposed to several warm treatments, then digestibility can decrease due to the development of disulphide bonds in the protein. Nafisa et al [27] found that boiling and tasting tree locusts enhances the content of tannins and phytates in full insects and decreases *in-vitro* protein digestibility in steamed and fried samples. Processing decreased protein digestibility, but some feed was still comparable to the reported prices for protein. The highest digestibility of brown locusts (85.67% of DM), green locusts (82.34% of DM), and fresh termites (90.49% of DM) were recorded in mink animals and compared well with the prices of plant protein sources [27–29].

### Grasshopper effect on nonruminant animal digestibility

The inclusion of GHM into the diet of chickens has enhanced feed conversion and protein digestibility. Adding GHM to the diet did not change the meat’s physical properties, but the sensory properties appear to improve with vision [30,31]. On the other hand, the following is an alternative to eating a fish meal with grasshoppers at rates of 0, 25, 50, 75, and 100 of in the broiler diet during the early and growing period. Replacing fish meal with grasshoppers significantly reduced feed intake, growth efficiency, and body production. During the growing phase of broilers, feed efficiency was not significantly affected [32]. In Nigeria, researchers studying broilers (1 to 28 days) replaced desert GHM with fish meal; replacing 50% fish protein with GHM (1.7% in diet) resulted in increased weight gain, feed intake, and feed conversion ratios [33].

In addition, fresh, toasted, freshly dried, and toasted dry samples generally showed decreases in all vitamins. Two types of insects had significantly lower riboflavin contents (p≤0.05) after toasting and solar drying compared to fresh samples. Five minutes of toasting at 150°C resulted in a 34% decrease in riboflavin material compared to fresh specimens [28]. However, subsequent sun-drying of the toasted sample at 30°C resulted in a significant loss of riboflavin c (64%) compared to fresh dried samples (46%), and processed grasshoppers showed a considerable decrease (p≤0.05) in vitamin B_3_ and ascorbic acid [28,34].

### Black soldier fly larvae chemical composition and nutritive value

Black soldier fly larvae (BSFL) originate in cattle, pig, and poultry manure, but they can mature on organic waste materials, such as catsup, coffee beans pulp, caribou, and vegetables [35]. Adult BSFs have a wasp-like shape, are blue or black, and have two translucent “windows” on the first part of the abdomen. Adult BSFs range from 15 to 20 mm in length.

Different researchers have reported different nutrients in feeds made from BSF larvae (Table 1). On average, BSFL consist of DM (27.40%), CP (56.10%), CF (23.20%), ash (9.85%), Ca (2.14%), P (1.15%), Mg (0.39%), K (1.35 %), Zn (13.10 mg/kg), Cu (11.20 mg/kg), Mn (23.20 mg/kg), and Fe (20.40 mg/kg) on the basis of DM. BSFL (also known as eating BSF larvae, BSF pre-poppy food, and BSF worm food) are used directly or are dried, chopped, and ground into shapes. The DM substance in fresh BSFL is much higher (34.9% to 44.9%), which makes BSFL easier and less expensive than other fresh products. On average, BSFL consists of 41.1% to 43.6% CP, 15.0% to 34.8% EE, 7.0% to 10% CF, ash 14.6% to 28.4%, and 5,278.49 kcal/kg GE, based on DM [36,37]. BSFL larvae are high in Ca (5% to 8%) and P (0.6% to 1.5%). Furthermore, the mineral profile contains Cu (6.0 mg/kg), Fe (0.14% to 14%), Mn (246 mg/kg), Mg (0.39%), sodium (Na, 0.13%), K (0.69%), and Zn (108 mg/kg) [34,35]. According to Cullere et al [35], the primary EAA were alanine (Ala), glutamic acid (Glu), leucine (Leu), and valine (Val), were affluent in BSF larvae. De Marco et al [36] found smaller amounts of arginine (Arg), histidine (Hist), lysine (Lys), and methionine (Meth) in the BSFL diets than in the previous investigation, but isoleucine (Isoleu), phenylalanine (Phy), and threonine (Thar) animal protein reversed the position.

The FA synthesis of BSFL depends on the synthesis of FA in the diet. Black soldier fly larvae fed on cow dung contained lauric acid (21%), oleic acid (32%), omega (30.2%), and palmitic acid (16%). The FA ratio of BSFL was an offer to fish 43%, 11%, 12%, and 3%, and 50% cow dung [5]. The whole lipid substance improved from 21% to 30% DM. Raising BSFL on cow fertilizer and increase 22% fish weight within 24 hours of their parasite is enough for adequate improvement of polyunsaturated FAs, especially eicosapentaenoic acid, and docosahexaenoic acids [37].

### Black soldier fly larvae effect on nutrient digestibility in ruminants

Possible sources of insect protein for cattle feed include Jamaican field crickets, BSFL, and mealworms. Jayanegara et al [37] used these three types of insects to feed Friesian Holstein cattle. They reported that the insect feeds usually contain 40% DM than Jamaican field cricket and also insect feed was marked with higher fiber content than SBM. In insect feed, black soldiers found significantly higher neutral detergent fiber (NDF), and acid detergent fiber (ADF) in larvae concerning *in vitro* rumen fermentation facial appearance and digestibility of insect and SBMs revealed similar total volatile FA concentrations, acid detergent insoluble CP ratio were higher and lower *in-vitro* DM digestibility and *in-vitro* organic matter (OM) digestibility in insect feed than SBM. Insect feed produces less gas due to higher fiber and EE contents than SBM. In these *in vitro* systems, gas is formed primarily through carbohydrate fermentation, and EE’s involvement in gas production is insignificant. The high level of NH_3_ in the incubation of Jamaican field cricket relates to its high CP content. The elimination of proteolysis and physiological degradation by proteolytic microorganisms in the rumen form NH_3_ [38]. The formation of NH_3_ in the rumen is not entirely dependent on CP concentration and includes other factors, such as CP portion, protein degradation rate, passage rate, use of NH_3_ to form microbial proteins, and renal absorption of NH_3_ through the rumen wall blood flow [39]. Animals given feeds containing all insects excreted much less methane than those fed SBM, and the decreased insect digestibility resulted in low H_2_ production, which is an important substrate for the production of methanogenesis [40].

### Black soldier fly larvae effect on nutrient digestibility in non-ruminants

Black soldier fly larvae are ahead of significance in the diet of livestock ability to accelerate products through the low-cost agricultural industry in high-protein biomass. The required nutritional content of BSFL for use as protein-rich biomass in feed for pork, fish, and poultry raised for food has not been met. In addition, insect digestibility depends not just on the insect species and breeding substrate but also on processing techniques and regulations (e.g., time and temperature) [39]. The standard DM digestibility of BSFL was 71%±2.81% of DM when fed to leopard geckos and 26%±9.9% of DM for mountain chicken frogs. Significant digestive benefits were also found because of the Cu, molybdenum, Mg, K, protein, Na, Fe, and Zn. Some differences in indigestion may be associated with the different percentages of larvae among studies. When raising quail [40], three diets were tested as controls, 10% nonstandard BSFL feed (soybean oil 28.4% and SBM 16.1% substitute) and 15% BFSL feed (soybean oil 99.9% and SBM substitute 24.8%). The quail showed the same body weight gain, feed intake, feed conversion ratio, and mortality rates in every experimental group. The evident nutrient digestibility (i.e., CP, DM, EE, OM, and starch) was similar in the three groups, except for EE, which had the maximum digestibility at 92.9% for the control and 89.6% for the 15% BSFL feed. Upon examination the quail showed no preference for the control. Furthermore, the breast meat weight and production did not differ between the control group and those fed 15% BSFL 15% feed.

### Housefly (*Musca domestica*) chemical composition and nutritive value

The housefly (HF) head has eyes, antennae, and mouthparts. Housefly increase food digestion by using saliva deposited from the mouthparts. Antennas provide housefly with their primary source of smell and often vary among males and females. The widespread housefly larvae (HFL) can breed on cattle, pig, and poultry manures, and HFL can be raised on public waste material. The life-cycle of the HF has multiple stages: eggs; larvae of first, second, and third instar; pupa; and adult. The duration from egg to adult is approximately 7 to 10 days in warm temperatures and 40 to 49 days in cooler weather [41].

The HFL is ready to create maggots of food. They are stored for fast reproductive tempo, high food cost, and simple to process and durable utilize [42]. Housefly larvae have high amounts of energy, protein, micronutrients (e.g., Cu, Fe, Zn), EAA, and FA. Housefly larvae are inexpensive, have high-quality nutritional value, and are less troublesome to make from other sources of animal protein. In general, HFL meals have high amounts of Lys, Thr, and Met and can be added to low-protein cereals and legume-based diets for livestock [8,43]. Different researchers have reported different nutrient levels for feeds using HFL. Table 1 lists the average nutritive values of HFL. On average, Housefly larvae include ash (6.25%), DM (83.47%), CP (33.29%), CF (6.20%), Ca (0.49%), P (1.09%), Mg (0.23%), K (1.27%), Zn (10.39 mg/kg), Cu (32.40 mg/kg), Mn (42.50) mg/kg, and Fe (47.50 mg/kg) on DM basis.

According to Hwangbo et al [44], HFL is rich in CP (63.99% of DM) and EE (24.31% of DM). The CP and EE contents can differ because of drying techniques and the maggots’ ages. The CP content decreases and the EE content increases with age. According to Makkar et al [5] and Khan [8], HFL feed included ash (10.68% of DM), CP (60.38% of DM), EE (14.08% of DM), and GE (4,800.80 kcal/kg), and HFL included ash (7.73% of DM), CP (76.23% of DM), EE (14.39% of DM), and GE (4,877.23 kcal/kg). They noted that HF pupas and larvae have apparent metabolic energies of 3,398.77 kcal/kg and 3,618.51 kcal/kg, respectively. The larvae showed high Ca, P, and metabolic energy (4,140 kcal/kg) compared to SBM (2,250 kcal/kg). Linoleic acid was found at concentrations of 26.3% to 36.3% of the whole fat in feeds containing HF pupae and larvae, respectively. In addition, maggots contain a high percentage of palmitoleic acid, and HF pupae are high in essential FAs such as oleic acid and linoleic acid. Maggots also had higher AAs and EAAs than SBM. In particular, the maggots had high levels of EAAs, such as Lies, Arg, Fi, Trap, and Val, but low concentrations of meth and sesame. Thus, meth will need to be offered in conjunction with diets containing maggots. The contents of CP, Phy, Lys, and Meth were higher in HF pupae than they were in mealworms (MWs). In terms of protein value, larvae were similar to bone and meat meal, as well as fishmeal (FM), and better than SBM. The maggots had more pronounced digestion of protein (98.5%) and EAA (94.8%) than SBM diets [45]. It was famous that the maggot has the highest CP content in raw egg concentrates (45.8%) and EE (19.3%) compared to non-bee rations (39.5% and 19.1%, respectively). The maggots food people who are involved in chopped mangoes have a higher amount of ash (7.1%) maggots were eaten with the raw egg attracting the maximum CP (6.1%). The maximum CP value was observed 48 hours after harvest (58.0%). Most were EE and CP 120 hours harvest (24.6 and 7.6 respectively) were observed [46].

### Housefly larvae effect on nutrient digestibility in ruminants

Bosch et al [45] found that nitrogen was in 83.2% and 84.3% of OM *in vitro* digestion capabilities and HF fly papaya, respectively. The proximate investigation showed as EE 13.9% and 12.9% with total tract digestibility of 93.9% and 97.9%, CP 59.9% and 75.9% with whole tract digestibility of 68.9% and 78.9%, CF 7.9% and 14.9%, with total tract digestibility of 61.9% and 57.9%, GE 4,800.8 kcal/kg and 4,877.2 kcal/kg, and apparent metabolizable energy (ME) 3,398.77 kcal/kg and 3,618.51 kcal/kg respectively [46]. Nutrition analysis Housefly larvae feed were shown to have comparable values to most high protein feed ingredients. The larval diet consisted of 60% proteins with a balanced AA profile and 20% fat with 57% monounsaturated FA, and 39% saturated FA. The larvae were short of food any significant amount of omega-3 FA [45,46].

### Housefly larvae effect on nutrient digestibility in non-ruminants

There are two studies of apparent digestion of dried HF feed that were tested in broiler chickens. Hwangbo et al [44] fed four weeks older broilers are fed 30% dry HF larvae or a diet for 7 day with SBM and three weeks older broilers are fed corn meal diet which contains 50% dry HFL feed. The consequences show that the apparent importance in the first study, the concentration of CP (98.5%) for HF larvae, was higher than in the 2nd study (69.5%) and the final study showed that CP fecal digestion was higher for HF pupae than for larvae. According to Pieterse and Pretorius [47] found that the significantly better visible fecal digestibility standards for individual AAs than CP. There is requiring evaluating the nutrient digestibility of insects as a ration component, which is a requirement for preparing insect-containing diets. The AAs analysis exposed AAs sympathetic with high Lys concentration but slightly low meth concentration. The ratio among Arg 0.67%, Lys 0.9%, Isoleu 10.68%, Leu 0.6%, respectively were found in larvae and pupae [48].

### Mealworm larvae (*Tenebrio molitor*) chemical composition and the nutritive value

Mealworm is grown on dried and cooked waste material in various sketches from fruits, grains, and vegetables. They are vegetarians but are usually fed in flour with wheat bran or soybean flour [5]. A mealworm is a beetle-like form of edible worms, a type of bean sprouts, dark-colored beetles. Like holometabolic insects, they go through four stages of life: the egg, the larva, the pupa, and the adult. Larvae typically measure about 2.5 cm or more, while adults typically range between 1.25 and 1.8 cm [49]. There are differences in the nutrients in the diet of mealworms reported by different researchers. The average nutritive value of mealworms was listed in Table 1. The average percentage of the mealworm consist of DM 94.6%, CP 55.83%, CF 25.19%, ash 4.84%, calcium 0.21%, phosphorous 1.06%, Mg 0.3%, K 1.12%, Na 0.21%, Zn 138.2 mg/kg, Cu 19.4 mg/kg, Mn 5.7 mg/kg, Fe 71.50 mg/kg of DM basis (Table 1).

The scientific literature on the nutritional synthesis of mealworm larvae shows different variations depending on the diet, climatic situation, and stage of maturity. Recently, Bovera et al [49,50] match up to the chemical composition and AAs profile of mealworm larvae with SBM diet and it has been reported that the ratio of 51.93%, CP 21.57% EE, and 7.20% ADF in mealworm larvae then SBM diet CP 44.51%, EE 1.84% and ADF 4.79% respectively. Both sources of the protein had a different mixture of EAAs, and this was especially evident for apparent for methionine and cystinone (SBM showed 3.27 times more content than MW) other than Arg (1.70%), Isol (1.75%), and Lys (1.68%) and higher in SBM diet hist (1.19%), Leu (1.25%), Thr (1.26%), and Val (1.10%), while the content of tryp was higher in MW larvae than in SBM diet [51]. Every supplementary AA had sufficient levels in MW larvae and can meet broiler requirements [47]. Currently, Hussein et al [6] reported that 44.9% CP and 33.9% EAAs and almost all EAAs particularly Lys (4.51%) and Meth (1.34%) were relatively high in MW.

According to Ravzanaadii et al [51] who found that the mealworm larvae contained a very small concentration of Ca (434.59 mg/kg) and a higher concentration of P (7,060 mg/kg). The ratio of Ca: P is not sufficient for poultry production especially for chickens, however, such problems can be solved by feeding WMs with a Ca prepared diet for 1 or 2 day [5]. The micro-mineral report was established to be Cu 13.27 mg/kg, Fe 66.87 mg/kg, and Zn 104.28 mg/kg, and also significant long-chain FAs synthesis was detected in larval fed with the highest component of linoleic acid 30.23%, oleic acid 43.17%, and palmitic acid 15.79%, respectively [37].

### Mealworm larvae effect on nutrient digestibility in ruminants

Insect-eating, black soldiers fly, Jamaican field cricket and mealworms are protein supplements for livestock. According to Jayanegara et al [37] reported that the results of the above insects fed to Friesian Holstein cows showed that the ratio of acid detergent insoluble CP and neutral detergent insoluble CP were slightly lower as compared to SBM. All insect feeds had lower *in-vitro* DM and OM digestibility than those in SBM (p<0.05). However, all insect feed produced less methane than those of SBM at 12, 24, and 48 h (p<0.05). Very limited data have been reported on the use of insect meal in ruminants [39,52,53]. The use of dietary HF and MW fat larva has been reported not to affect the digestibility of lactating rabbits at low inclusion rates of 0.75% to 0.1.5%. However, the high-fat content (3% to 6%) of BSFL resulted in lowering the DM, OM, EE, and GE digestibility [52,53].

According to Gasco et al [53] who offered two kinds of insects (MW and HF) to rabbit, and as result, the control and experimental groups found no difference between feeding insect fat in terms of performance, disease, mortality, and blood metabolites. The increase in HF and MW fats did not affect the appearance DM digestibility, CP, EE, fiber fraction, and energy. The gut morphometric index and organ histopathology were not affected by the addition of house fly and mealworm fat diets. Similar results were reported by Gugołek et al [54] who showed supplementation of fish meal and MW meal had no considerable variation in the digestibility of DM, OM, total energy, total protein, and NDF between the treatments and the control group. However, it should be noted that total protein digestion was higher in groups control and mealworm than in the fish meal group [54]. An enzymatic method was used to analyze total protein digestibility of *Tenebrio molitor* and *Hermetia illucens in-vitro* digestion experiment. The authors found that the digestion of raw protein in dites was affected by the level of chitin. In both insect feed samples, total protein digestion was negatively associated with ADF and chitin content. However, the average chitin yield in mealworm larvae was at 4.92% of DM [55].

### Mealworm larvae effect on nutrient digestibility in non-ruminants

The yellow mealworm was found to have 91.5% and 91.3% *in vitro* and organic digestibility, respectively in comparison with the two insect larvae feedings (MW and BSF) due to nutrients digestibility and coefficient of total tract apparent digestibility of apparent ME and AAs digestibility coefficients for broilers [56]. There was no significant variation in all nutrients digestibility values except for the EE value and the value of MW was higher than of the BSF meals [8]. The MW larval hydrolysate increased digestibility of DM, CP, Lye, Meth, when compared to other nutritional supplements from using fish hydrolysate [8,57].

The dietary value of dry mealworm larvae directly resembled that of FM, making it a potentially smart alternative protein-rich feed component for the animal feed industry as compared to other nutritional supplements such as FM, meat meal, or chicken meal [58]. On the other hand, the small amounts of animal protein can be supplemented with dried mealworm larvae. The digestibility of CF was higher in chinchillas fed on mealworms. Both alternative diet sources of protein in the investigational diets improved the digestion of ADF and acid detergent lignin [59].

May researchers have measured the CP digestibility of some insect larvae [58–60]. Stein et al [61] reported that the apparent digestible diet of BSF larvae in male growing pigs was 76% parallel to SBM digestibility. Jin et al [60] have shown that with an increase in the concentration of dried mealworm in the feed, the overall digestibility of CP was significantly increased. Under this study, the digestibility was 92.1% when the level of dried mealworm in the diet was 4.5% and 93.0% when added at 6.0% [58].

## ECONOMICAL ASSESSMENT OF INSECT PROTEIN AS COMPARED TO OTHER PROTEIN SOURCES

The insect producers also need a scale to prove their credibility so that consumers can be confident about the quality of the product, stability, and safety. The price varies according to the price of the insect and there are currently improving insect manufacturers and production, which will have a huge impact on overall production costs. To be viable, insect protein products must have a live weight of 0.40 €/kg based on 35% DM. The up-to-date price of frozen dry edible insects for attractive fish is currently 3.70 €/kg and for testing rationale, a high-rank protein meal of BSF larvae was quoted at meal 20 €/kg (Protix Biosystems, Dongen, The Netherlands) [62]. In addition, the market value of commercially available maggots meal is 1.08 €/kg compared with protein-rich feed ingredients, worms are the most viable protein fish with a genuine price of 1.24 €/kg and are expected to increase soon. The up-to-date price of soybeans feed (CP >480 g/kg) is approximately 0.57 € [62,63]. Conversely, to make a good assessment among the insects and traditional protein sources, the nutritional value must be adjusted, for example, based on digestible protein or digested AAs. In order to be viable, insect manufacturers aim to reduce the cost good-quality protein.

Rising the size of insect breeding companies will further enhance efficiency and reduce the cost of insect protein. Some other potential for cost reduction are as follows: i) reduction in feed costs by increasing conversion of bio-waste products. ii) Rising the size of insect breeding companies and capable use of the building will reduce housing costs, e.g., by dropping energy use and improving heat exchange and ventilation. iii) Enhancing the productivity by advanced breeding and breeding techniques; iv) Developing the reaction extraction efficiency (insect fat and insect protein) [64]. In addition to reducing costs, rising product prices will help compete with insects. The valuable practical properties of insects have the potential to enhance the value of pests as feed ingredients. Organic farmers may also be involved in raising insects to enhance the permanence of animals in their fields. Insects are actually present in the natural habitat of insects [62–64]. In addition, farming of insects capable of biodegradation of organic wastes according to Čičková et al [65] and Van Huis [66] reiterated that BSF and HF production has been more potentially promising for feeding interventions.

Adjusting the prices of insecure insects based on their nutritional value would be the best feasible way to compare them with traditional proteins [63]. Based on this, the prices of insects were considered based on their protein, Lys, and Met mixture. The permanent model was experimental at the protein value, the lysine and methionine value was measured for all protein sources. The highest price per kg of protein, Lys, and Met was traced for BSF while SBM has cheaper prices. The estimated cost of an opportunity to replace SBM with insects depended on the price of protein, Lys, and similar protein value of SBM. The consequences specify that the cost of protein, Lys, and Met per kilogram of SBM replaced by BSF would cost for farmers 88.23, 92.43, and 142.52 €/kg, respectively. Similarly, replacing one kilogram of protein, Lys, and Met from SBM with HF maggot with cost an additional 3.98, 3.85, 3.85, and 6.73 €/kg, respectively [64,67]. In order to convert the equivalent amount of protein, Lys, and Met provided by SBM for MW, the farmer will have to bear an additional loss of 13.0, 11.15, and 15.02 €/kg, respectively. FM’s economic values were similar to those of HF maggot based on its cost and nutritional values. When compared to SBM, BSF, HF, maggot, and MW were not economically possible. However, it is possible to replace FM with HF from an economic point of view, although their actual digestibility needs to be considered properly when comparing their economic values [68].

On the fictitious expansion of commercial poultry farms (which spend € 1,000 per month on protein derived from SBM), which means to completely replace SBM with BSF, HF, or MW. The additional feed cost will be 88,230, 3,980, and 13,010 €, respectively. Consider that if farmers make all year round, it can cost a fortune to take over the field at a profit margin. Before insects can be adopted as an alternative to SBM and FM, both cost and nutritional value must be sustainable. To be competitive, the cost of insects should be reduced, 0.4 €/kg of direct weight based on 35% DM substances. This and many other possible strategies to improve insect competition were suggested by alternative sources of protein [62–64]. The Economic values of insects compared to other sources of proteins see in Table 2.

## CURRENT INSECT FARMING PRODUCTION STATUS

The various varieties of insects have been an ingredient of the diet for farm animals (e.g., pigs, poultry, and especially fish). Human utilization of insect-based food is attractive a more and more popular alternative source of protein and an estimated two billion communities currently eat insects or insect-based foods. In many fields, entomophagy is a native process. However, in Western countries, insect utilization tends to increase, but yet most people reject insects as food [69].

The edible pests are produced in three ways: deforestation, semi-breeding, and farming. According to Yen [68], moths represent 92% of the world’s insect supply, and semi-domestic insects make up 6%. This means that only 2% of the provided insects for, now, if we only consider those that are direct human use. Insect farming is a recent method of producing edible insects, especially in developed countries [70, 71]. According to Van Huis and Oonincx [70], who offered more information on the farming system, advantages, and disadvantages, insecticide is undoubtedly the majority productive and effective way to produce pests.

Some of the technological and administrative benefits come from raising insects more than most livestock substitutes for natural protein production. According to Mlcek et al [71] and Govorushko [72] summarizes the benefits associated with such farming: i) Insect farming requires less gap than conventional livestock. This requires imperfect investment costs (protein manufactured per unit); ii) Genocide is carried out with completely simple technology; iii) This type of farming can lead to faster returns on investment and faster financial returns; and iv) This farm is easy to manage and does not require in-depth education.

Insect farming has been developed worldwide for feed and food production, and it is becoming a vital business not simply in the tropics [73]. Insect farming for the feed industry has improved significantly in recent decades due to the natural potential of many livestock and fish methods to act as traditional feed. The implanted energy from the OM used to breed insects is completely changed into high-value edible protein for human and livestock feeding [74,75]. The result is a rapid worldwide growth in the insect feed market, which, according to Mordor Intelligence [76], was valued at 687.8 million USD in 2018, with an annual growth rate of 12% reaching a value of 1,396.4 million USD. In particular, until 2018, the insect feed market in Asia-Pacific conquered in response to enhance meat utilization. It is expected to be Europe’s fastest-growing market in the next few years. Analyzing the insect feed promote by animal category, aquaculture is the primary buyer (51% of the shares), while market share analysis shows the most scattered buyers with some transfer companies. (Altec Coupon, Intrafeed Corporation, Insect, Agro Protein, and Environ Flight) [68,75]. On the other hand, some micro-enterprises can do economically the use of easy skills and possibilities offered locally, in particular, insecticides are exploited in the aquaculture sector. This means that internationally, a lot of companies are concerned in the preparation of insect feed and food, some of which are concerned in the preparation of animal feed, focusing only on the production of insect feed [76]. Moreover, insects farming is more socio-economically and environmentally-friendly under low carbon-scenario with high feed utilization efficiency [15]. In this background, there are considerable sell opportunities and challenges for the insect feed and food sectors, such as profitable scale manufacture, high-quality standards, excessive quantities, low ecological impact, and the need for spirited prices [76].

According to Gahukar [77] wanted to explore the economic viability of insect farming as an alternative source of protein production. Gahukar pointed out that insect farming is not cheap at present. The main cost represents the raw material used to feed the insects and, today, the cost per feed produced is quite high (e.g., 18 for 25 kg feed for cricket breeding) [29]. There is a lack of economic literature on the expediency of introducing insect diets into animal feed. In particular, these results show that on the basis of bee food, i.e., BSF and HF are cheaper than FM (in this case, used for boiler breeding) [78]. The investment in livestock was projected at 25% higher come back due to the high feed conversion related to insect feed and the beneficial value of raw materials (the cost of insect feed is approximately 71% of fish and SBM) [5,8]. According to Arru et al [78] established that the use of insect feed in aquaculture, especially in the cultivation of European territorial waters, is derived from the yellow MW which is the most widely used for fish farming and the study was conducted in Italy and concluded that where the cost of fish is higher in Europe than in insect meal. The authors pointed out several rule implications in the study, signifying that large-scale pest production would promote various economic profits (such as income and job production) in rising countries [75].

## ADVANTAGE AND LIMITATION OF INSECTS USE IN ANIMAL DIETS

Insects are the potential source of alternative protein. Insect-based feed products may have a market like fish and SBM, which are currently the major ingredients in aquaculture and livestock feed formulas. Also, one kilogram of worm protein takes much less feed and soil of meat protein. However, the significant existing legislation and regulations need to be reviewed certainly this type of mini-livestock is permissible to enter the animal feed.

### Sustainable feed ingredients

There is a need to increase meat consumption in developing countries and thus to encourage the tapping of proteins into alternative feed sources. “Insects are gradually recognized as a better protein substitute use in animal feed”, reads a project briefing document, noting that many species are highly nutritious and their other sources of feed reduce the environmental impact of production.

### Digestibility of insect

Digestive capacity can be better assessed in *in-vivo* digestibility trials. Digestive imitation can be effective in digesting tolerance conditions (pepsin/HCL) and small intestine (buffer/pancreas). Therefore, it is functional to compare with the elements that are well described in the feed. For poultry, the *in-vivo* digestibility of insect diets in terms of AAs is 89% to 95%, which is dependent on AAs and is equivalent to FM. Housefly pupae performed similarly *in-vitro* digestion for OM and protein compared to fish meal and poultry meal.

### Low cost

A Young French company, Ynsect, has recognized an inexpensive, nutritious, and locally sourced alternative to soybeans as an important source of protein in animal feed. Earlier this year, a BSF, HF, MW, and silkworms were named among the mainly capable species for industrial feed production by the UN Food Agency. According to the FAO, proteins such as meat, fish meal, and soybean feed account for 60 to 70 percent of the price.

### Animal health benefits

According to the investigator, BSF protein derivatives can promote the health of pets and fish: “Aging is the major challenge in livestock”. Aging can accelerate the loss of free radicals in a livestock body, leading to widespread health disorders (such as locomotor disorders). Insect proteins, including livestock feed and water, are legal in Europe and are gaining traction. Insect proteins are especially suitable for young animals to eat, as young animals grow faster and develop their immune systems. It appears that the black solder fly protein derivative can help suppress free radical damage in livestock bodies. Aquatic organisms, on the other hand, are at constant risk of pathogenic bacteria attacks, resulting in the development of a variety of health conditions, including immunity, aging, and so on.

### Medical uses of insect

Bee venom stimulates apoptosis in rheumatoid synovial cells by reducing BCL2 expression and rising BAX and Caspase-3 expression. Bee venom stimulates apoptosis through caspase 3 activation in synovial fibroblasts in patients with rheumatoid arthritis.

The toxins found in bee venom can kill the human immunodeficiency virus (HIV). Bee venom contains melittin, which surrounds the HIV virus as well as other viruses. It is loaded with melittin nanoparticles that attack an integral part of the virus’s structure. Nanoparticles are easy to manufacture in large quantities for future clinical trials.

Maggot therapy deliberately launches sterile flying larvae (maggots) to selectively clean squashy tissue wounds. These assists stop the infection and also cures chronic wounds and ulcers. Wounds that have been healing for days and those that have been affected are better than those that have not healed. Maggots eat several chemicals that kill microbes, including allantoin, calcium carbonate, phenylacetic acid, proteolytic enzymes, phenylalanine, urea, and many more. Maggots were used by the Maya and Indigenous Australians for wound healing.

## REGULATORY OF INSECT USES BY THE GOVERNMENTS IN ASIA

Edible insect regulations safety policies and regulations about the edible pests should be significant to the governments of both developing and developed countries to make sure that consumers are provided with a reduced or risk-free supply of this type of food from farm to fork [79]. The utilization pattern of customers have changed significantly in recent times due to their growing understanding of the quality and rights of safe foods, and this undoubtedly applies to edible insects and their derivatives [80]. Thus, the article clarifies that insects should be marketed for human utilization, especially for human consumption. Those insect food products must comply with existing good manufacturing methods (CGMP) for processing, packaging and transportation. And to protect consumers, there should be a warning label for shellfish allergies [81].

In Western countries, food regulations pose a major difficulty to the use of insects in both feed and food. The European Food Safety Authority has declared that all insect products for human utilization will be considered ‘novel food’ and must be suggested for novel food authorization by 2018, with a transition period of 2 years in advance. Approved products will be allowed to remain in the market until 2020 [82]. However, quantities of European Union (EU) member states have their own legislation to address this need. There are similar laws around the approval of novel food in North America. In Canada, authoritarian approvals must come from the Canadian food inspection agency and health Canada, and the food and drug administration in the United States, as well as the association of American feed control officers for feed ingredients definition committee [80].

In Asia, Thailand, the world’s main breeder of cricket, is working on creating the first set of guidelines. The Thai Government Agency for Agricultural Products is expected to issue guidelines for good agricultural practices for the growth of cricket by the end of 2017. The initial set of these rule for Government Agency for Agricultural was made public by Khon Kaen University [81]. There is a average of cricket farming in Thailand. The standards include farm ingredients, animal health, food, environment, water, and record keeping principles. The goal is to produce high-quality cricket that is safe for customers. Food, water, should not be spoiled, equipment should be clean and hygienic, and all chemicals should be used according to the instructions [83]. The expansion of Thai cricket farming values has been linked to cricket exports, mostly access to EU markets Food is administered by the Food and Drug Administration Thailand. In aquaculture, Thai companies want to replace unsafe fish meat with insects. Thailand also has a huge broiler meat and pork industry with a potential for insect-based feed. Thailand has food quality standards for all kinds of animals, but insect feed standards do not yet exist [84].

The ministry of public health (MPH) is presently the reliable organization that regulates insect production and utilization in Thailand. All pest-processed products that are sold and exported in local markets must be approved by law through the Food and Drug Administration under the MPH. Insect breeders must apply for a license, and a Food and Drug Administration inspector oversees the production position, as well as sampling and monitoring products for suitable hygiene standards on a regular basis. There are no specific standards for food as insects. Thus, they are treated like any other food product under the Food Act (Food and Drug Administration Thailand 2014) of B.E.2522 (1979). This is probably recognized to the fact that there is a long history of entomophagy in Thailand as well as in the bigger region of Southeast Asia, and contradictory food standards have not been developed to protect consumers [81].

Feng et al [85] reveled that edible insects have been used in Chinese medicine and as food for more than 2000 years. Currently, 324 species from 11 orders are reported in China. In China, insects are a common feed and food component in several areas, but are not yet declared in the food law. One exception, though, is the silkworm pupae, which were added to the Health Ministry’s list of approved foods in 2014. China is the world’s biggest manufacturer of silk and silkworms are accessible in large quantities. They are also exported for food consumption, such as in Thailand [80].

The South Korean government launched a process in 2011 to legalize some edible insects. Following this initial process, in 2016, the Korean Food and Drug Administration classified cricket and food worms as common foods without restriction. Other bugs are expected to be added to the eligibility list soon [80,81].

In Singapore, they did not move to give it a green light. The Singapore Food Agency (SFA) has not yet approved the significance of insects as food for human utilization. The various food safety problems may be related to pest utilization, and the SFA will review whether it is safe to use before importing or selling. The companies intending to importance insects for human utilization (in Singapore) must submit an application with supporting food safety evidence [79–81].

## CONCLUSION

Insects have been important component of the natural environment, significantly attribute to the diversity and usefulness of nature and mankind. Among many inter-connections, the insect characterization and their nutritional values have been currently provocative especially the quantity and quality of protein as well as macro-minerals and bioactive substances contained enormously in most of the insects. Furthermore, current information pertaining to modern cultivation and farming have exhibited the practical potential for productive cultivation for both uses in human consumption and as a protein source in animal diets to replace conventional source of protein such as SBM and fish meal. Nevertheless, more proactive investigations using insect protein for animal feeding for both nonruminants and ruminants are warranted to elucidate additional data.

## Figures and Tables

**Table 1:** Types of economical insect and their chemical composition and nutritive value

S.No	Insects species	Percentage (%)									Milligram per kilogram (mg/kg					References
	
		DM	CP	CF	Ash	Ca	P	Mg	K	Na	S	Zn	Cu	Mn	Fe	
1	*Grasshopper*	35.00	50.50	15.30	6.40	146.0	153.0	56.40	3.44	ND	ND	0.04	0.01	1.40	0.06	[2,25,27]
2	Black soldier fly larvae	27.40	56.10	23.20	9.85	2.14	1.15	0.39	1.35	0.13	27.04	13.10	11.20	23.20	20.40	[2,34,35, 36,37,42,43]
3	Housefly larvae	83.47	33.29	6.20	6.25	0.49	1.09	0.23	1.27	0.54	ND	10.39	32.40	42.50	47.50	[6,42,48]
4	Mealworm larvae	94.60	55.83	25.19	4.84	0.21	1.06	0.30	1.12	0.21	ND	138.2	19.40	05.70	71.50	[54,55]

DM, dry matter; CP, crude protein; CF, crude fiber; Ash; Ca, calcium; P, phosphorous; Mg, magnesium; K, potassium; Na, sodium; Zn, zinc; Cu, copper; Mn, manganese; Fe, iron.

**Table 2:** The economical values of insects compared to other sources of protein

Potential source	Housefly maggot	Black soldier fly	Mealworm	Fishmeal	Soybean meal
CP (%)	50.4	42.1	52.8	75.4	52.00
Lysine (%)	6.1	6.6	5.4	7.5	6.3
Methionine (%)	2.2	2.1	1.5	2.8	1.3
PPR (€/kg)	1.08	20	3.7	1.24	0.2
PP (€/kg)	2.14	47.51	7.01	1.64	0.54
PL (€/kg)	0.13	3.14	0.38	0.12	0.03
PM (€/kg)	0.05	1.00	0.11	0.05	0.01
PP to PP SBM^1)^	3.98	88.23	13.01	3.05	1.00
PL to PL SBM^1)^	3.85	92.23	11.15	3.64	1.00
PM to PM SBM^1)^	6.73	142.52	15.02	6.58	1.00
References	[2,5,70,71]	[70,71]	[2,5,70,71]	[2,5,70,71]	[2,5,70,71]

PPR, product price; PP, protein price; PL, price of lysine; PM, price of methionine; PB, protix biosystems; AP, Agri protein.

1) PP to PP SBM, price of replacing 1 kg of protein from SBM with other protein sources; PL to PL SBM, cost of replacing 1 kg of lysine from SBM with lysine from other protein sources; PM to PM SBM, cost of replacing 1 kg of methionine from SBM with methionine from other protein sources.

## References

[1] Da-Silva Lucas AJ, de Oliveira LM, da Rocha M, Prentice C (2020). Edible insects: An alternative of nutritional, functional and bioactive compounds. Food Chemist.

[2] Makkar HPS (2018). Review: Feed demand landscape and implications of food-not feed strategy for food security and climate change. Animal.

[3] Meneguz M, Schiavone A, Gai F (2018). Effect of rearing substrate on growth performance, waste reduction efficiency and chemical composition of black soldier fly (Hermetia illucens) larvae. J Sci Food Agric.

[4] FAO (2009). The State of Food and Agriculture: Livestock in the Balance.

[5] Makkar HPS, Tran G, Heuzé V, Ankers P (2014). State-of-the-art on use of insects as animal feed. Anim Feed Sci Technol.

[6] Hussein M, Pillai VV, Goddard JM (2017). Sustainable production of housefly (Musca domestica) larvae as a protein-rich feed ingredient by utilizing cattle manure. PLoS ONE.

[7] Miech P, Lindberg JE, Berggren Å, Chhay T, Jansson A (2017). Apparent faecal digestibility and nitrogen retention in piglets fed whole and peeled Cambodian field cricket meal. J Insect Food Feed.

[8] Khan SH (2018). Recent advances in role of insects as alternative protein source in poultry nutrition. J Appl Anim Res.

[9] Schiavone A, Dabbou S, De Marco M (2018). Black soldier fly larva fat inclusion in finisher broiler chicken diet as an alternative fat source. Animal.

[10] Secci G, Moniello G, Gasco L, Bovera F, Parisi G (2018). Barbary partridge meat quality as affected by Hermetia illucens and Tenebrio molitor larva meals in feeds. Food Res Int.

[11] Gasco L, Finke M, van Huis A (2018). Can diets containing insects promote animal health?. J Insect Food Feed.

[12] Onsongo VOI, Osuga IM, Gachuiri CK (2018). Insects for income generation through animal feed: effect of dietary replacement of soybean and fish meal with black soldier fly meal on broiler growth and economic performance. J Econ Entomol.

[13] Sealey WM, Gaylord TG, Barrows FT (2011). Sensory Analysis of Rainbow Trout, Oncorhynchus mykiss, Fed Enriched Black Soldier Fly Prepupae, Hermetia illucens. J World Aquac Soc.

[14] Barroso FG, de Haro C, Sánchez-Muros MJ, Venegas E, Martínez-Sánchez A, Pérez-Bañón C (2014). The potential of various insect species for use as food for fish. Aquaculture.

[15] Van Huis A (2013). Potential of insects as food and feed in assuring food security. Annu Rev Entomol.

[16] Ahmed S, Al Baki MA, Lee J, Seo DY, Lee D, Kim Y (2021). The first report of prostacyclin and its physiological roles in insects. Gen Comp Endocrinol.

[17] Khusro M, Andrew NR, Nicholas A (2012). Insects as poultry feed: a scoping study for poultry production systems in Australia. World's Poult Sci J.

[18] Zhang L, Lecoq M, Latchininsky A, Hunter D (2019). Locust and grasshopper management. Annu Rev Entomol.

[19] Ojewola GS, Eburuaja AS, Okoye FC, Lawal AS, Akinmutimi AH (2003). Effect of inclusion of grasshopper meal on performance nutrient utilization and organ of broiler chicken. J Sustain Agric Environ.

[20] Wang D, Zhai SW, Zhang CX, Zhang Q, Chena H (2007). Nutrition value of the Chinese grasshopper Acrida cinerea (Thunberg) for broilers. Anim Feed Sci Technol.

[21] Ghosh S, Parimalendu H, Dipak KM (2016). Evaluation of nutrient quality of a short horned grasshopper Oxya hyla hyla Serville (Orthoptera: Acrididae) in search of new protein source. J Entomol Zool Stud.

[22] Ssepuuya G, Mukisa IM, Nakimbugwe D (2017). Nutritional composition, quality, and shelf stability of processed Ruspolia nitidula (edible grasshoppers). Food Sci Nutr.

[23] Straub P, Tanga CM, Osuga I, Windisch W, Subramanian S (2019). Experimental feeding studies with crickets and locusts on the use of feed mixtures composed of storable feed materials commonly used in livestock production. Anim Feed Sci Technol.

[24] Patton RS, Chandler PT (1975). In vivo digestibility evaluation of chitinous materials. J Dairy Sci.

[25] Kinyuru JN, Kenji GM, Njoroge SM, Ayieko M (2010). Effect of processing methods on the in vitro protein digestibility and vitamin content of edible winged termite (Macrotermes subhylanus) and grasshopper (Ruspolia differens). Food Bioproc Tech.

[26] Opstvedt J, Nygard E, Samuelsen TA, Venturini G, Luzzana U, Mundheim H (2003). Effect on protein digestibility of different processing conditions in the production of fish mealand fish feed. J Sci Food Agric.

[27] Nafisa ME, Hassan SY, Hamed AB, Hassan MM, Elfadil EB (2008). Nutritional evaluation and physiochemical properties of boiled and fried tree locust. Pakistan J Nutr.

[28] Nginya ES, Ondiek JO, King’ori AM, Nduko JM (2019). Evaluation of grasshoppers as a protein source for improved indigenous chicken growers. Breas.

[29] Moula N, Detilleux J (2019). A meta-analysis of the effects of insects in feed on poultry growth performances. Animals.

[30] Brah N, Houndonougbo FM, Issa S (2018). Grasshopper meal (Ornithacris cavroisi) in broiler diets in Niger: Bioeconomic performance. Int J Poult Sci.

[31] Falade KO, Omojola BS (2010). Effect of Processing Methods on physical, chemical, rheological, and sensory properties of Okra (Abelmoschus esculentus). Food Bioproc Technol.

[32] Patterson PH, Acar N, Ferguson AD, Trimble LD, Sciubba HB, Koutsos EA (2021). The impact of dietary Black Soldier Fly larvae oil and meal on laying hen performance and egg quality. Poult Sci.

[33] Arango Gutierrez GP, Vergara Ruiz RA, Mejia Velez H (2004). Compositional microbiological and protein digestibility analysis of larval meal of Hermetia illucens (Diptera:Stratiomyidae) at Angelopolis-Antioquia Colombia. Rev Fac Nac Agron Medellin.

[34] St-Hilaire S, Sheppard C, Tomberlin JK (2007). Fly prepupae as a feedstuff for rainbow trout Oncorhynchus mykiss. J World Aquac Soc.

[35] Cullere M, Tasoniero G, Giaccone V (2016). Black soldier fly as dietary protein source for broiler quails: apparent digestibility, excreta microbial load, feed choice, performance, carcass and meat traits. Animal.

[36] De Marco M, Martínez S, Hernandez F (2015). Nutritional value of two insect larval meals (Tenebrio molitor and Hermetia illucens) for broiler chickens: Apparent nutrient digestibility, apparent ileal amino acid digestibility and apparent metabolizable energy. Anim Feed Sci Technol.

[37] Jayanegara A, Yantina N, Novandri B, Laconi EB, Nahrowi N, Ridla M (2017). Evaluation of some insects as potential feed ingredients for ruminants: chemical composition, in vitro rumen fermentation and methane emissions. J Indones Trop Anim Agric.

[38] Owens FN, Qi S, Sapienza AD (2014). Applied protein nutrition of ruminants current status and future directions. Anim Sci.

[39] Jayanegara A, Dewi SP, Ridla M (2016). Nutrient Content, Protein Fractionation, and Utilization of Some Beans as Potential Alternatives to Soybean for Ruminant Feeding. Med Pet.

[40] Jayanegara AG, Goel HP, Makkar S, Becker K (2015). Divergence between purified hydrolysable and condensed tannin effects on methane emission rumen fermentation and microbial population in vitro. Anim Feed Sci Technol.

[41] Boushy AR (1991). House-fly pupae as poultry manure converters for animal feed: a review. Bioresour Technol.

[42] Campbell M, Ortuño J, Stratakos AC (2020). Impact of thermal and high-pressure treatments on the microbiological quality and in vitro digestibility of black soldier fly (Hermetia illucens) Larvae. Animal.

[43] Moreki JC, Tiroesele B, Chiripasi SC (2012). Prospects of utilizing insects as alternative sources of protein in poultry diets in Botswana. J Anim Sci Adv.

[44] Hwangbo J, Hong EC, Jang A (2009). Utilization of house fly-maggots, a feed supplement in the production of broiler chickens. J Environ Biol.

[45] Bosch G, Zhang S, Oonincx DGAB, Hendriks WH (2014). Protein quality of insects as potential ingredients for dog and cat foods. J Nutr Sci.

[46] Ukanwoko AI, Olalekan OA (2015). Effects of source and time of harvest on the proximate composition of maggot (Musca Domestica) larva meal. Int J Livest Res.

[47] Pieterse E, Pretorius Q (2013). Nutritional evaluation of dried larvae and pupae meal of the housefly (Musca domestica) using chemical- and broiler-based biological assays. Anim Prod Sci.

[48] Rumpold BA, Oliver KS (2013). Potential and challenges of insects as an innovative source for food and feed production. Innov Food Sci Emerg Technol.

[49] Bovera F, Loponte R, Marono S (2016). Use of Tenebrio molitor larvae meal as protein source in broiler diet: Effect on growth performance, nutrient digestibility, and carcass and meat traits. J Anim Sci.

[50] Józefiak D, Józefiak A, Kierończyk B (2016). Insects a natural nutrient source for poultry a review. Ann Anim Sci.

[51] Ravzanaadii N, Kim SH, Choi WH, Hong SJ, Kim NJ (2012). Nutritional value of mealworm, Tenebrio molitor as food source. Int J Indust Entomol.

[52] Shah AA, Liu Z, Qian C, Wu J, Sultana N, Zhong X (2020). Potential effect of the microbial fermented feed utilization on physicochemical traits, antioxidant enzyme and trace mineral analysis in rabbit meat. J Anim Physiol Anim Nutr.

[53] Gasco L, Dabbou S, Trocino A (2019). Effect of dietary supplementation with insect fats on growth performance, digestive efficiency and health of rabbits. J Anim Sci Biotechnol.

[54] Gugołek A, Strychalski J, Juśkiewicz J, Żary-Sikorska E (2020). The effect of fish and mealworm larvae meals as alternative dietary protein sources on nutrient digestibility and gastrointestinal function in Chinchilla lanigera. Exp Anim.

[55] Marono S, Piccolo G, Loponte R (2015). In vitro crude protein digestibility of tenebrio molitor and hermetia illucens insect meals and its correlation with chemical composition traits. Ital J Anim Sci.

[56] Song YS, Kim MW, Moon C (2018). Extraction of chitin and chitosan from larval exuvium and whole body of edible mealworm, Tenebrio molitor. Entomol Res.

[57] Matin N, Utterback P, Parsons CM (2021). True metabolizable energy and amino acid digestibility in black soldier fly larvae meals, cricket meal, and mealworms using a precision-fed rooster assay. Poult Sci.

[58] Yoo JS, Cho KH, Hong JS (2019). Nutrient ileal digestibility evaluation of dried mealworm (Tenebrio molitor) larvae compared to three animal protein by-products in growing pigs. Asian-Australas J Anim Sci.

[59] Schiavone A, De Marco M, Rotolo L Nutrient digestibility of Hermetia illucens and Tenebrio molitor meal in broiler chickens. https://iris.unito.it/handle/2318/158360#.YQS1AUBRXIU.

[60] Jin XH, Heo PS, Hong JS, Kim NJ, Kim YY (2016). Supplementation of dried mealworm (Tenebrio molitor larva) on growth performance, nutrient digestibility and blood profiles in weaning pigs. Asian-Australas J Anim Sci.

[61] Stein HH, Kim SW, Nielsen TT (2001). Standardized ileal protein and amino acid digestibility by growing pigs and sows. J Anim Sci.

[62] Veldkamp T, Bosch G (2015). Insects: a protein-rich feed ingredient in pig and poultry diets. Anim Front.

[63] Veldkamp T, Van Duinkerken G, van Huis A (2012). Insects as a sustainable feed ingredient in pig and poultry diets: a feasibility study Insecten als duurzame diervoedergrondstof in varkens-en pluimveevoeders: eenhaalbaarheidsstudie (No 638).

[64] Malla N, Opeyemi AJ (2018). Prospects of insects as alternative protein source: broiler chicken and growing pigs. Report in Sustainable Animal Nutrition and Feeding.

[65] Čičková H, Newton GL, Lacy RC, Kozánek M (2015). The use of fly larvae for organic waste treatment. J Waste Manag.

[66] Van Huis A (2020). Insects as food and feed, a new emerging agricultural sector: a review. J Insects Food Feed.

[67] Madau FA, Arru B, Furesi R, Pulina P (2020). Insect farming for feed and food production from a circular business model perspective. Sustainability.

[68] Yen AL (2015). Insects as food and feed in the Asia Pacific region: Current perspectives and future directions. J Insects Food Feed.

[69] Oonincx DGAB, de Boer IJM (2012). Environmental impact of the production of mealworms as a protein source for humans - a life cycle assessment. PLoS ONE.

[70] Van Huis A, Oonincx DGAB (2017). The environmental sustainability of insects as food and feed. A review. Agron Sustain Dev.

[71] Mlcek J, Rop O, Borkovcova M, Bednarova MA (2014). A Comprehensive look at the possibilities of edible insects as food in Europe a review. Pol J Food Nutr Sci.

[72] Govorushko S (2019). Global status of insects as food and feed source: a review. Trends Food Sci Technol.

[73] Kim TK, Yong HI, Kim YB, Kim HW, Choi YS (2019). Edible insects as a protein source: a review of public perception, processing technology, and research trends. Food Sci Anim Resour.

[74] Derrien C, Boccuni A, Halloran A, Flore R, Vantomme P, Roos N (2018). Current status of the insect producing industry in Europe. Edible insects in sustainable food systems.

[75] Diener S, Solano NMS, Gutiérrez FR, Zurbrügg C, Tockner K (2011). Biological treatment of municipal organic waste using black soldier fly larvae. Waste Biomass Valorization.

[76] (2019). Mordor Intelligence Insect Feed Market Growth Trends and Forecasts 2020–2025.

[77] Gahukar RT, Dossey AT, Morales-Ramos JA, Rojas MG (2016). Edible insects farming: efficiency and impact on family livelihood, food security, and environment compared with livestock and crops. Insects as sustainable food ingredients.

[78] Arru B, Furesi R, Gasco L, Madau FA, Pulina P (2019). The introduction of insect meal into fish diet: the first economic analysis on European sea bass farming. Sustainability.

[79] Imathiu S (2020). Benefits and food safety concerns associated with consumption of edible insects. NFS J.

[80] Lähteenmäki-Uutela A, Marimuthu SB, Meijer N (2021). Regulations on insects as food and feed: a global comparison. J Insects Food Feed.

[81] Hanboonsong Y, Jamjanya T, Durst PB (2013). Six-legged livestock: edible insect farming, collecting and marketing in Thailand.

[82] IPIFF (International Platform Insects for Food & Feed) EU Legislation.

[83] Halloran A, Vantomme P, Hanboonsong Y, Ekesi S (2015). Regulating edible insects: the challenge of addressing food security, nature conservation, and the erosion of traditional food culture. Food Secur.

[84] Preteseille N, Deguerry A, Reverberi M, Weigel T, Halloran A, Flore R, Vantomme P, Roos N (2018). Insects in Thailand: national leadership and regional development, from standards to regulations through association. Edible insects in sustainable food systems.

[85] Feng Y, Chen XM, Zhao M (2018). Edible insects in China: Utilization and prospects. Insect Sci.

